# New Aradidae from Ecuador (Hemiptera, Heteroptera, Aradidae)

**DOI:** 10.3897/zookeys.319.4755

**Published:** 2013-07-30

**Authors:** Ernst Heiss

**Affiliations:** 1 Research Entomologist, Tiroler Landesmuseum, Josef Schraffl Strasse 2a, A 6020 Innsbruck, Austria

**Keywords:** Hemiptera, Heteroptera, Aradidae, Mezirinae, Carventinae, new genus, new species, apterous, micropterous, Ecuador

## Abstract

As an addition to the presently poorly known aradid fauna of Ecuador, 3 new genera and 4 new species are described: *Osellaptera setifera* **gen. n.**, **sp. n.**; *Kormilevia ecuadoriana* **sp. n.** both belonging to Mezirinae; and Carventinae *Cotopaxicoris cruciatus* **gen. n.**, **sp. n.** and *Onorecoris piceus* **gen. n.**, **sp. n.** An updated key is provided for all species of the Neotropical genus *Kormilevia* Usinger & Matsuda, 1959.

## Introduction

The aradid fauna of Ecuador was first assembled and catalogued by Froeschner 1981 reporting 15 species, belonging to the subfamilies Aneurinae Douglas & Scott, 1865 (2 spp.) and Mezirinae Oshanin 1908 (13 spp.). This reflects very poorly on the known flat bug fauna of this country, which is expected much more diverse and numerous because of its different biotops and suitable habitats. Later additions were recorded in Kormilev and Froeschner’s synonymic list of the “Flat Bugs of the World” in 1987 and again updated by Coscaron and Contreras in their “Catalog of Aradidae for the Neotropical Region” in 2012.

A small lot of Aradidae from Central Ecuador in the authors collection contained – not unexpectedly – several new taxa. Although only single specimens are available, their striking morphological differences from other Neotropical Aradidae justify the erection of new genera for them. They are illustrated and described below as *Osellaptera setifera* gen. n., sp. n.; *Kormilevia ecuadoriana* sp. n. both belonging to the subfamily Mezirinae; and *Cotopaxicoris cruciatus* gen. n., sp. n. and *Onorecoris piceus* gen. n., sp. n. of the subfamily Carventinae. An updated key is proposed for all species of the Neotropical genus *Kormilevia* Usinger & Matsuda, 1959.

## Material and methods

The specimens upon which the descriptions are based are dry-mounted and preserved in the collection of the author at the Tiroler Landesmuseum, Innsbruck, Austria (CEHI). As specimens of these apterous and pilose new taxa were mostly covered by detritus or incrustations, they were cleaned by treatment in 10% KOH for the study of abdominal structures. Photos were taken with an Olympus SZX 10 binocular microscope and a Olympus E 3 digital camera, processed with Helicon Focus 4.3 software, using Adobe Photoshop and Lightroom 2.3.

Measurements were taken with an eyepiece micrometer (20 units = 1 mm).

Abbreviations used: deltg = dorsal external laterotergite (connexivum); mtg = abdominal median tergite; pe-angles = posteroexterior angles (of deltg); vltg = ventral laterotergite. When citing the text on the labels of a pin attached to the specimens / separates the lines and // different labels.

## Taxonomy

### Subfamily Mezirinae Oshanin 1908

#### Osellaptera gen. n.

urn:lsid:zoobank.org:act:3B994BCD-645A-4233-96F4-B7CA4CCA2BCD

http://species-id.net/wiki/Osellaptera

##### Type species:

*Osellaptera setifera* sp. n.

##### Diagnosis.

Although superficially resembling the habitus of *Mystilocoris pubescens* Usinger & Matsuda, 1959 from Colombia, the new species cannot be placed in any of the apterous or micropterous Neotropical Mezirinae genera; here therefore *Osellaptera* gen. n. is proposed. It is distinguished from *Mystilocoris pubescens* (the only species of this genus) by its micropterous condition (apterous im *Mystilocoris*), more slender antennae, head as long as wide (about 1.5x wider in *Mystilocoris*), different structure and fusion of thorax and abdomen, and position of spiracles II-IV ventral, V sublateral and hardly visible from above, VI and VI sublateral on a prominent tubercle and visible, VIII lateral (II-VI ventral, VII sublateral and visible, VIII lateral), and the shape of the pygophore.

##### Description.

Small sized micropterous Mezirinae; body oval, laterally constricted at metanotum, attenuated posteriorly, surface of body with deep punctures and long pubescence on carinate structures; legs and antennae beset with long setae; colouration cinnamoneous, legs and antennae are lighter.

Head. Triangular as wide as long, clypeus narrow reaching ½ of antennal segment I; antenniferous lobes short; antennae slender twice as long as width of head, segment I longest, II+III shorter, IV shortest; eyes stalked; postocular lobes converging to constricted neck.

Pronotum. Distinctly wider than long, lateral margins converging anteriorly with a vertically reflexed triangular expansion, disk with 2 ovate callosities depressed between them.

Mesonotum. Strongly transverse, median elevated ridge fused to that of metanotum and mtg I+II; lateral sclerites callous delimited laterally by flap-like structures these regarded as reduced remnants of wingpads.

Metanotum. Fused to mtg I+II, lateral sclerites oval and callous; surface of mtg I+II punctured delimited posteriorly by carinae expanding laterally from median ridge.

Abdomen. The median ridge of thorax continues along the tergal plate of mtg III-VI and highest on mtg IV; lateral sclerites with subrectangular punctured depressions; deltg II+III fused, anteriorly constricted, reaching mesonotum; lateral margins of deltg II-VI reflexed, those of deltg II-IV laterally expanded and pilose.

Venter. Metathoracic scent gland canal curved anteriorly and upward, not visible from above; spiracles II-IV ventral, V sublateral and barely visible from above, VI and VII sublateral on a prominent tubercle and fairly visible, VIII lateral and visible from above.

Legs. Long and slender, claws with thin pulvilli.

Etymology. It is a pleasure to dedicate this conspicuous new flat bug genus to my friend Giuseppe Osella (Verona), appreciating his long time friendship and generosity donating to me Aradidae from his collection trips, and recognizing his important contributions to various matters of coleopterology.

#### Osellaptera setifera sp. n.

urn:lsid:zoobank.org:act:53337094-CD36-4238-A232-E4386BA21613

http://species-id.net/wiki/Osellaptera_setifera

Fig. 1

##### Holotype male

labelled: Ecuador, Cotopaxi / Otonga (foresta nublada) / 2000m 23–30 VII 2004 / G. Osella leg. This specimen is designated as holotype and labelled accordingly.

##### Description.

Holotype male, micropterous body surface deeply punctured interrupted with smooth carinae bearing dense pilosity; colouration cinnamomeous, appendages lighter with long dense erect setae curved on apices.

Head. As wide as long (26/26); pilose clypeus produced and narrowly rounded anteriorly reaching 1/3 of antennal segment I; genae thin and adherent as long as clypeus; antenniferous lobes short, apex recurved apically; antennae twice as long as width of head (52/26), segment I club-shaped and longest, II and III shorter and cylindrical thickened apically, IV shortest, clavate with pilose apex; length of antennal segments I/II/III/IV = 18/12/14/8; eyes stalked directed anterolaterally; postocular lobes sinuately converging to constricted neck; vertex with a median elevation laterally separated from smooth oval callosities by deep grooves; rostrum arising from a slit-like atrium, shorter than head, rostral groove with carinate borders.

Pronotum. About 2.8× as wide as long (31/11); lateral margins converging anteriorly with a vertically reflexed triangular expansion, disk with 2 ovate callosities deeply depressed between them, anterolateral angles slightly produced and rounded; anterior margin concave, ring like; posterior margin convex.

Mesonotum. Strongly transverse, 3.35× as wide across wingpads as long, consisting of a median ridge fused to that of metanotum and mtg I+II and of lateral oval callosities delimited by an inclined pilose carina followed by flap-like wingpads produced over lateral margins of abdomen.

Metanotum. Fused to mtg I+II these visible as transverse punctured depressions, posteriorly delimited by curved pilose carinae which are connected to median ridge, lateral sclerites of metanotum punctured and callous, posteriorly sloping to mtg I without a separating suture.

Abdomen. Tergal plate of mtg III-VI medially elevated and connected anteriorly to thoracic ridge, highest on mtg IV, lateral sclerites with subrectangular punctured depressions; triangular deltg II+III fused, produced and constricted anteriorly, reaching lateral margin of mesonotum; deltg II-IV laterally expanded, their pe-angles produced and beset with long setae; tergite VII with 2 (1+1) sublateral smooth callosities, strongly medially raised for the reception of the large pygophore, this pyriform projecting posteriorly; paratergites VIII small, much shorter than pygophore, surface with short yellowish pilosity; the cleft visible between tergite VII and anterior margin of pygophore shows triangular apices of parameres; the single male was not dissected for furtherstudy of the latter.

Venter. Metathoracic scent gland canal curved anteriorly and upward, not visible from above; spiracles II-IV ventral, V sublateral and barely visible from above, VI and VII sublateral on a prominent tubercle and fairly visible, VIII lateral and visible from above.

Legs. Long and slender, claws hook-like with thin pulvilli, with protibial comb.

Measurements. Length 5.4mm (incl. cleft pygophore); width of abdomen at apex of deltg II 2.1mm; across tergite III 2.6mm, across tergite VII 2.3mm; length / width of pygophore 0.45/0.75mm; length of antennae 2.6mm.

Etymology. The name refers to the dense setae covering legs and antennae and most body parts.

**Figures 1–2.**
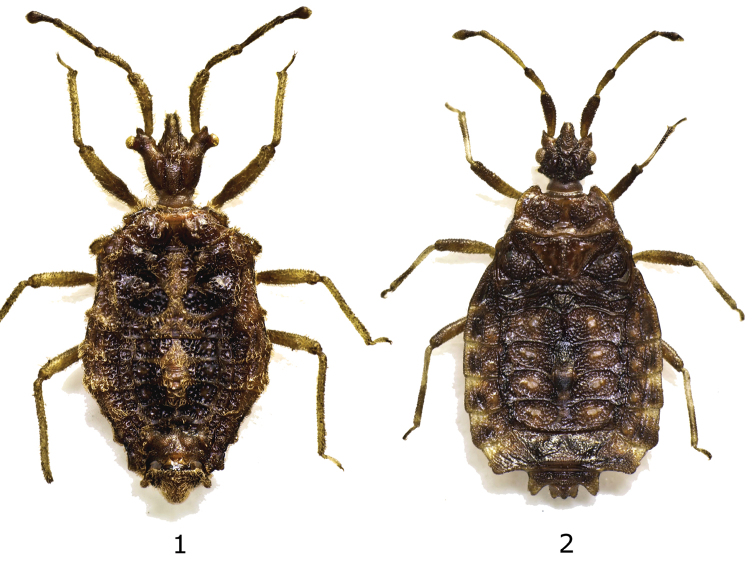
**1** *Osellaptera setifera* gen. n., sp. n., holotype male dorsal view **2** *Kormilevia ecuadoriana* sp. n., holotype female dorsal view.

#### Genus Kormilevia

Usinger & Matsuda 1959

http://species-id.net/wiki/Kormilevia

##### Remarks.

The micropterous genus *Kormilevia* Usinger & Matsuda, 1959 was erected for *setifera* Usinger & Matsuda, 1959 from Brazil. These authors recognized that species described as *Acaricoris dureti* Kormilev, 1953a from Argentina, or as *Pictinus plaumanni* Kormilev, 1953b and *Pictinus montrouzieri* Kormilev, 1953b, both from Brazil, belong to *Kormilevia* and were transferred there giving a key to the 4 then known species. They were however uncertain about the taxonomic position of *Acaricoris teresopolitanus* Wygodzinsky, 1948 and did not include this in the key. Kormilev 1963 described the next species *aberrans* sp. n. from Colombia and 1964 *gerali* sp. n. from Brazil, and presents a key to 6 species including *teresopolitana* but omitting his *aberrans*.

As the comparative notes of Kormilev’s descriptions are partly insufficient, the important structures of the mesonotum are not described or illustrated (e.g., *aberrans*, *gerali*) and the position of spiracles used in Kormilev’s 1964 key are contradictory to the descriptions (*plaumanni*, *montrouzieri*), a comparison of taxa is uncertain and needs a revision based on the types. Here all available data from the literature were assembled and a tentative new key for all 8 taxa is presented below. For *plaumanni* and *montrouzieri* original material was available for study (ex. coll. Plaumann).

All *Kormilevia* species are micropterous, although not always recognized as such, sharing small nonfunctional wingpads that enable only a very limited range of distribution. It can be assumed that they are therefore endemic to the region of origin. Because of biogeographical considerations – the closest record to the Ecuadorian locality is that of *aberrans* from Bogota, Colombia, which lies about 800km north – and distinct characters, *Kormilevia ecuatoriana* sp. n. is described here although only a single female is available.

**Key to species of Kormilevia**

**Table:** 

1 (2)	Spiracles II-VI ventral, remote from lateral margin, VII+VIII lateral and visible from above; only female holotype known (described as A *caricoris*), 4.8mm, Fig. 7 (Brazil)	*eresopolitana* (Wygodzinsky) 1948
2 (1)	Spiracles II-IV ventral, V-VI sublateral or lateral and barely visible from above, VII-VIII lateral	3
3 (4)	Antennae long, about 2.6x as long as width of head, antennal segment IV as long as II, only female holotype known, of larger size, 5.3mm, Fig. 2 (Ecuador, Cotopaxi)	*ecuatoriana* sp. n.
4 (3)	Antennae shorter, 2.03-2.28x as long as width of head, antennal segment IV longer than II, of smaller size (except *aberrans* =4.55-5.3mm)	5
5 (6)	Eyes as long as distance from anterior margin of eye to apex of antenniferous lobes, holotype male 3.65mm, paratype female 4.0mm, Fig. 8 (Brazil, Est. do Rio)	*etifera* Usinger & Matsuda 1959
6 (9)	Eyes large, longer than distance from anterior margin of eye to apex of antenniferous lobes	7
7 (8)	Length of antennae 2.28x as long as width of head, head about as long as wide,smaller species, only male holotype known, 3.5mm, Fig. 6 (Argentinia, Iguazu)	*dureti* (Kormilev) 1953
8 (7)	Antennae 2.12x as long as width of head, head distinctly wider than long, larger species, holotype male 4.55mm, paratype female 5.32mm (Colombia, Bogota)	*aberrans* Kormilev 1963
9 (6)	Eyes smaller, shorter than antenniferous lobes	10
10 (11)	Spiracles II-IV ventral, in both sexes, V sublateral and barely visible from above, VI-VIII lateral and distinctly visible from above, holotype male 4.0mm, paratype female 4.5mm (Brazil, Sta. Catarina)	*gerali* Kormilev 1964
11 (10)	Spiracles II-IV ventral, in females V sublateral and barely visible from above, VI-VIII lateral and visible, in male V-VIII lateral and visible from above	12
12 (13)	Scutellum like mesonotum with a median V-shaped posteriorly raised ridge and a median groove at base, male pygophore posteriorly obtuse, holotype male 3.9mm, paratype female 4.25mm, Figs 3, 4 (Brazil, Sta. Catarina)	*montrouzieri* (Kormilev) 1953
13 (12)	Scutellum like mesonotum with a parallel ridge reaching from base to apex with an indistinct median suture, male pygophore posteriorly conical, holotype male 3.9mm, paratype female 4.4mm, Fig. 5 (Brazil, Sta. Catarina)	*plaumanni* (Kormilev) 1953

**Figures 3-8.**
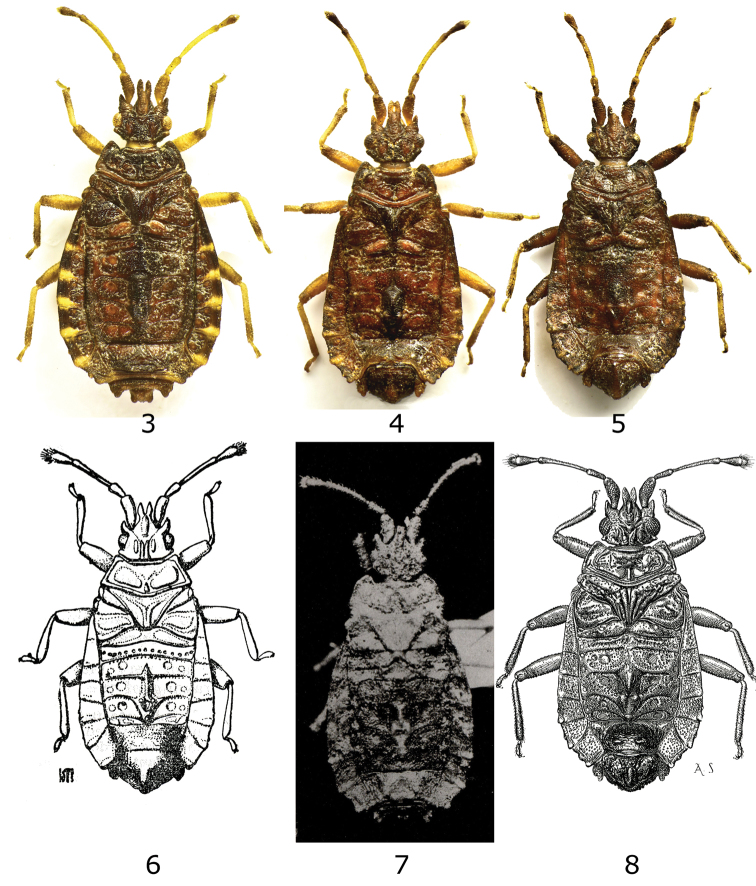
*Kormilevia* species. **3** *Kormilevia montrouzieri* female from Sta. Catarina, Brazil **4** *Kormilevia montrouzieri* male from same locality **5** *Kormilevia plaumanni* male from Sta. Catarina, Brazil **6** *Kormilevia dureti*, male holotype (from Kormilev 1953) **7** *Kormilevia teresopolitana*, female holotype (from Wygodzinsky 1948) **8** *Kormilevia setifera*, holotype male (from Usinger and Matsuda 1959).

#### Kormilevia ecuadoriana sp. n.

urn:lsid:zoobank.org:act:35441D85-FADC-4EB8-8EAF-EBE72B208B46

http://species-id.net/wiki/Kormilevia_ecuadoriana

Fig. 2

##### Holotype female

labelled: Ecuador 2008 / legg. Baviera, Belló / Osella & Poliano // ECU-Cotopaxi / Otonga – Galapagos / m 1620, 5 VIII 2008 / 00°23.962'S, 58°56.720'W. The specimen is designated as holotype and labelled accordingly. CEHI.

##### Diagnosis.

The new species differs from all 7 species assigned to date to the genus *Kormilevia* Usinger & Matsuda, 1959 catalogued by Kormilev and Froeschner 1987 and can be recognized by the long antennae and other characters mentioned in the key.

##### Description.

Small sized micropterous Mezirinae; body oval, abdomen dilated posteriorly, surface of body granulate and rugose, lateral margins with short setae; antennae and legs beset with fine setigerous tubercles. Colouration of body reddish brown, pronotum except oval callosities, smooth part of scutellum, apodemal impressions of tergal plate and anterolateral angles of deltg II-VII and apices of tergites VII-X yellowish; antennae yellowish, apical half of segment I, apex of II and III and basal half of IV darker brown, legs yellowish, femora and tibiae brown on apical half.

Head. Slightly longer than width across eyes (20/17.5); clypeus conical; genae thin and adherent, as long as clypeus, reaching about 1/3 of antennal segment I; antenniferous lobes short with acute apex; antennae 2.62× as long as width of head (46/17.5), segment I thickest, II and IV thinner and shortest, dilated apically, III thinnest and longest, cylindrical, IV with pilose apex; length of antennal segments I/II/III/IV = 13/8/17/8; eyes granular inserted in head, their length shorter than antenniferous lobes (9/10); postocular lobes notched behind eyes followed by a round lateral tubercle not reaching outer margin of eyes, then straight, converging to constricted collar; vertex with irregular rugosities separated laterally by a deep groove from oval callosities; rostrum arising from a slit-like atrium, shorter than head.

Pronotum. More than twice as wide as long (31/12); sinuate lateral margins emarginate and carinate converging anteriorly, anterolateral angles narrowly rounded, produced over anterior margin; posterior margin carinate, slightly convex; disk smooth depressed at middle, flanked by 2 (+1) oval granulate callosities.

Mesonotum. Consisting of a triangular scutellum-like plate with smooth surface and anterior and lateral margins carinate and a median granulate carina, anterolaterally delimited by small rounded wing pads, lateral polygonal sclerites with a median oval granulate callosity; metathoracic scent gland visible from above posterior to wing pads.

Metanotum. Transverse, surface rugose with 2 (1+1) submedian ovate callosities, depressed between them; posterior margin nearly straight, separated from fused tergites I+II by a distinct suture. Tergites I+II fused, posterior margin delimited by a bisinuate suture, raised medially.

Abdomen. Tergal plate flat, consisting of mediotergites III-VI laterally with large oval depressions, medially elevated on tergites IV and V; deltg II-VII slightly reflexed, their lateral margin subparallel at middle attenuated anteriorly and posteriorly, their surface longitudinally carinate on outer half; deltg II triangular not fused to deltg III, deltg II-VII separated by sutures; pe-angles of deltg VI slightly rounded, of deltg VII produced posteriorly over straight posterior margin of tergite VII; tergite VIII bilobate, visible tergites IX and X tricuspidate.

Venter. Spiracles II-IV ventral, V sublateral but barely visible from above, VI-VIII lateral and visible from above.

Legs. Long and straight, femora moderately incrassate medially, tarsi two-segmented, claws with pulvilli and a long median setiform parempodium.

Measurements. Length 5.3mm; width of mesonotum across wing pads 1.75mm; scutellum length / width 0.6/1.45mm; width of abdomen across tergites IV and V 2.85mm.

##### Etymology.

Named after Ecuador, the country of origin.

### Subfamily Carventinae Usinger, 1950

#### Cotopaxicoris gen. n.

urn:lsid:zoobank.org:act:02E9A006-655E-4EAF-A28D-D9A8D482740F

http://species-id.net/wiki/Cotopaxicoris

##### Type species:

*Cotopaxicoris cruciatus* sp. n.

##### Diagnosis.

The combination of characters: general habitus, long antennae, stalked eyes, pro-and mesonotum separated by sutures, metanotum fused to mtg I+II and to abdominal tergal plate, long pilosity on body, and appendages and the micropterous condition. This combination of characters is not shared by any apterous or micropterous Carventinae recorded from mainland South and Mesoamerica (*Aparilocoris* Kormilev, 1983; *Dihybogaster* Kormilev, 1953b; *Glyptocoris* Harris & Drake, 1944; *Kolpodaptera* Usinger & Matsuda, 1959; *Peggicoris* Drake, 1956, *Reeceicus* Drake, 1956). It stands also apart and shows no resemblances to genera described from the Caribbean Islands.

##### Description.

Micropterous; body subrectangular strongly attenuated anteriorly; surface of head and body with deep punctures, the elevated structures and lateral margins of head and body as well as of legs and antennae beset with fringe-like yellowish setae; colouration light brown, head darker, tibiae lighter.

Head. Distinctly wider than long, clypeus short, genae adherent shorter than clypeus; antenniferous lobes short diverging anteriorly; antennae about 2.5× as long as width of head; segment I longest, those following shorter and thinner; eyes stalked; postocular lobes converging posteriorly; rostrum arising from an open atrium as long as head.

Pronotum. Subrectangular about 3× as wide as long; lateral margins with rounded reflexed carinate paranota, posteriorly delimited by a notch followed by a laterally produced knob-like process; surface of disk with a median carina and rugose lateral sclerites; posterior margin carinate and convex, separated by a distinct suture from mesonotum.

Mesonotum. Strongly transverse, about 4x as wide as long; surface with a median ridge which continues throughout thorax and abdomen, laterally flanked by oval smooth depressions followed by rugose sclerites, these delimited laterally by basally elevated flap-like structures representing reduced wingpads; suture separating meso- and metanotum recognizeable lateral of median ridge, where it is marked only by a thin indistinct suture.

Metanotum. Fused to mtg I+II and tergal plate; continuous median ridge widened posteriorly then forming a cross-like ridge on mtg I+II; lateral sclerites with rugose callosities, depressed anteriorly; surface of mtg I+II deeply punctured.

Abdomen. Tergal plate fused to mtg I+II, median ridge narrower on mtg III continuing and raised posteriorly, highest on mtg IV-V; surface laterally with oval punctured depressions; deltg II+III fused, laterally expanded, an inclined carina marking posteror margin of deltg II; pe-angles of deltg V and VII roundly expanded.

Venter. Spiracles I-IV ventral remote from lateral margin, V and VI sublateral but visible from above, VII-VIII lateral and visible.

Legs. Long and slender, tarsi with long thin pulvilli.

##### Etymology.

Named after the Cotopaxi region, the second highest mountain in Ecuador, where this interesting new taxon was collected.

#### Cotopaxicoris cruciatus sp. n.

urn:lsid:zoobank.org:act:272A0E55-EC1E-48D9-BEAC-04EC9102047C

http://species-id.net/wiki/Cotopaxicoris_cruciatus

Fig. 9

##### Holotype male

labelled: Ecuador (Cotopaxi) 2008 / legg. Baviera, Belló / Osella & Pogliano // Vaglio bosque / nublado e\o sotto / legno e\o tronchi. CEHI. This specimen is designated as holotype and labelled accordingly.

##### Description.

Holotype male, micropterous; body surface deeply punctured with fringe-like pilosity on lateral margins of head, body, and carinate elevations, legs and antennae with fine erect setae; colouration light brown with darker head and lighter tibiae.

Head. Wider than long (27/24); clypeus short subparallel, genae adherent not reaching apex of clypeus; antenniferous lobes diverging anteriorly, apices narrowly rounded; antennae 2.48x as long as width of head (67/27); segment I longest and thickest, moderately incrassate along apical 2/3, II and III shorter and thinner, cylindrical, IV shortest spindle-shaped with pilose apex; length of antennal segments I/II/III/IV = 23/13/19/12; eyes stalked directed anterolaterally; postocular lobes roundly converging toward constricted neck densely beset with long erect setae with curved apices; vertex medially elevated with two rows of setae, laterally with 2 (1+1) large oval rugose callosities; rostrum arising from a slit-like atrium, as long as head, lateral margins of rostral groove carinate.

Pronotum. Subrectangular, 3× as wide across rounded lateral margins as long (33.5/11); lateral margins with rounded reflexed carinate paranota, posteriorly delimited by a notch followed posterolaterally by a laterally produced knob-like process; surface of disk with median carina and rugose lateral sclerites; posterior margin carinate and convex, separated from mesonotum by distinct suture.

Mesonotum. Strongly transverse, 4.5× as wide across wingpads as long at middle (45/10); consisting of two transverse laterally rounded sclerites lateral of median moderately elevated ridge, surface of sclerites with an oval smooth depression adjacent to median posteriorly widening ridge followed by rugose callosities; all margins carinate, anterolateral angles triangularly raised to level of adjacent knob-like process of pronotum, posterolaterally produced into oval flap-like expansions, these representing reduced wingpads; suture separating meso- and metanotum only developed lateral of median ridge, upon which the transverse fusion line is indistinct and barely discernible.

Metanotum. Fused to mtg I+II and tergal plate; continuous median ridge widened posteriorly then forming a cross-like elevated ridge on mtg I+II; lateral sclerites with rugose callosities, depressed anteriorly; surface of mtg I+II deeply punctured.

Abdomen. Tergal plate of abdominal mtg III-VI fused to mtg I+II, the median ridge narrower on mtg III continuing, widening and raised posteriorly, highest on abdominal scent gland at posterior margin of mtg IV-V; with oval punctured depressions laterally; lateral margins subparallel at deltg III+IV, dilated at deltg II, V and VI; deltg II+III fused, laterally expanded, an inclined carina marks the posteror margin of deltg II; pe-angles of deltg V and VII rounded expanded; tergite VII medially raised for reception of globose pygophore, this wider than long with a conical median elevation; paratergites VIII cylindrical produced over pygophore; the single male was not dissected for the study of parameres.

Venter. Spiracles I-IV ventral, remote from lateral margin, V and VI sublateral but visible from above, VII-VIII lateral and visible.

Legs. Long and slender, femora moderately incrassate, tibiae straight, tarsi with long thin pulvilli.

Measurements. Length 5.8mm; width of abdomen across tergite III 3.1mm, IV 3.15mm, V 3.2mm; width/length of pygophore 0.75/0.3mm; length of antennae 3.35mm.

**Figures 9–10.**
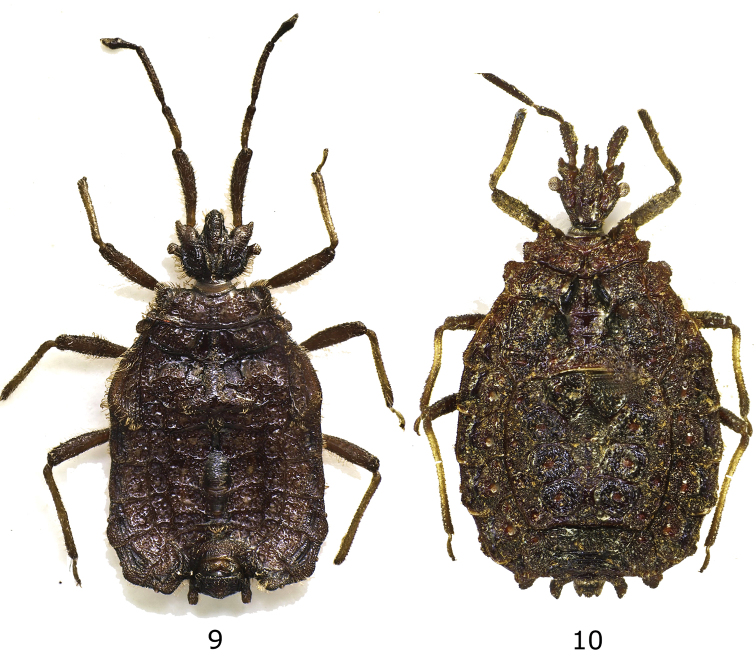
**9** *Cotopaxicoris cruciatus* gen. n., sp. n., holotype male dorsal view **10** *Onorecoris piceus* gen. n., sp. n., holotype female dorsal view.

##### Etymology.

Refers to the cross-like elevated ridge on the thorax.

#### Onorecoris gen. n.

urn:lsid:zoobank.org:act:FD590891-3410-449E-9961-33FC189561CC

http://species-id.net/wiki/Onorecoris

##### Type species:

*Onorecoris piceus* sp. n.

##### Diagnosis.

As in *Cotopaxicoris*, the assemblage of characters: general habitus, antennae twice as long as width of head, stalked eyes, a pentagonal fused median scerite on meso- and metanotum are not shared by apterous Carventinae recorded from South and Mesoamerica as reported for *Cotopaxicoris* description. Therefore, a new genus *Onorecoris* gen. n. is erected for *Onorecoris piceus* sp. n.

##### Description.

Apterous female; body oval attenuated anteriorly, surface of head and body rugose and tuberculate, legs and antennae beset with stiff hairs bearing tubercles; colouration piceous, tibiae yellowish except base and apex.

Head. Slightly longer than width across eyes; clypeus narrow, flanked by anteriorly produced genae reaching about ½ of antennal segment I; antenniferous lobes wide, apex narrowly rounded; antennae about twice as long as width of head, antennal segment III longest, I and II shorter, IV fusiform; eyes slightly stalked; postocular lobes converging uniformly to narrow neck region; rostrum arising from slit-like atrium, shorter than head.

Pronotum. About 3× as wide as long, lateral margins angulate at humeri then triangularly projecting anteriorly, produced over anterior margin; disk with median carina flanked by rugose callosities; posterior margin separated from mesonotum by a deep furrow.

Mesonotum. With median posteriorly widening and moderately elevated pentagonal ridge and lateral subrectangular sclerites with rugose surface, their lateral margins rounded, produced laterally; separated from metanotum along lateral sclerites by deep grooves, median ridge fused and continuing on metanotum and mtg I+II where its structure disappears.

Metanotum. Lateral sclerites separated from continuous median ridge by deep depressions, their surface deeply punctured and callous, posteriorly completely fused to mtg I+II; lateral margins with a small round expansion.

Abdomen. Tergal plate roundly elevated at middle, highest on mtg IV-V, lateral parts with oval punctured depressions these laterally delimited by carinate structures; lateral margins rounded with partly visible rims of dorsally reflexed vltg II-VII these increase posteriorly; pe-angles of deltg II-VII with a distinct dorsal tubercle; deltg II-VII separated by sutures, triangular deltg II anteriorly reaching to metanotum.

Venter. Surface rugose and punctured, spiracles II-IV ventral, V sublateral and visible from above, VI and VII on sublateral tubercles of reflexed vltg VI and VII and visible from above, VIII dorsolateral.

Legs. Long and slender, tibiae medially curved, claws with thin pulvilli.

Etymology. It is a pleasure to dedicate this interesting new flat bug genus to my friend Giovanni Onore (Quito), versatile and successful entomologist in Ecuador who made the species available for study.

#### Onorecoris piceus sp. n.

urn:lsid:zoobank.org:act:411698E8-6CA4-4023-81BF-20E4C2E085CB

http://species-id.net/wiki/Onorecoris_piceus

Fig. 10

##### Holotype female

labelled: Ecuador, Loja 7 Uritujinga 2800m / 19 Dec. 1997 G.Onore. CEHI. The specimen is damaged by a pinhole on the abdomen and lacks the right antennal segments II-IV. It was cleaned and remounted by the author. This specimen is designated as holotype and labelled accordingly.

##### Description.

Medium-sized apterous Carventinae, body broadly ovate, attenuated anteriorly; surface rather flat with rugosities and punctures; colouration piceous.

Head. Longer than wide (23/22.5, incl. neck 25/22.5); clypeus narrow, raised with a round tubercle subapically; genae thin, produced over apex of clypeus reaching about ½ of antennal segment I, antenniferous lobes diverging laterally, apex with a round tubercle; antennae 2.09× as long as width of head (47/22.5), segment I thickened on anterior ¾ densely beset with tubercles bearing stiff hairs, II shortest, III longest, IV fusiform with pilose apex; length of antennal segments I/II/III/IV = 12.5/8/18/8.5; eyes slightly stalked; postocular lobes uniformly converging to constricted neck; vertex with a median ridge, this posterolaterally with 2 (1+1) elevated round tubercles, separated from lateral oval callosities by deep grooves.

Pronotum. Strongly transverse, more than 3× as wide as long (35/11); lateral margins angularly produced on humeri then triangularly projecting anteriorly, longer than collar; disk with a V-shaped median sclerite anteriorly followed posteriorly by small median triangular ridge separated from oval callosities by deep grooves; posterior margin convex, transverse suture separates the mesonotum.

Mesonotum. Distinctly wider than long (45/10), consisting of a median posteriorly widening and moderately elevated pentagonal ridge and lateral subrectangular sclerites with rugose surface, their lateral margins rounded, produced laterally; separated from metanotum along lateral sclerites by deep grooves, median ridge fused, continuing on metanotum and mtg I+II where its structure is obliterated.

Metanotum. About 3.5× as wide as length including fused mtg I+II (52/15); lateral sclerites separated from continuous median ridge by deep depressions, their surface deeply punctured and callous, posteriorly completely fused to mtg I+II; lateral margins with a small round expansion.

Abdomen. Lateral and anterior margins of tergal plate convex, posterior margin straight; its surface rather flat with moderately rounded elevation on mtg IV-V, highest at scent gland scar IV-V; lateral parts with oval punctured depressions on mtg IV-VI, those of mtg III larger, directed anteriorly, their lateral margins delimited by carinae, these enlarged on anterolateral angles; deltg II-VII separated by sutures, triangular deltg II anteriorly reaching metanotum; pe-angles of deltg II-VII slightly reflexed, rounded, with larger dorsal, granulate tubercle; dorsally reflexed margins of vltg II-VII partly visible from above as lateral rims which increase in size from deltg II-VI forming triangular tubercle on deltg VII; tergite VII with a median elevated ridge, tergite VIII bilobate, visible apices of tergites IX and X tricuspidate, as long as posteriorly produced paratergites VIII.

Venter. Spiracles II-IV ventral, V sublateral and visible from above, VI and VII on sublateral tubercles of reflexed vltg VI and VII and visible from above, VIII dorsolateral¸sternites separated by transverse sutures, surface rugose and punctured,

Legs. Long and slender, femora cylindrical, tibiae medially curved, tarsi bisegmented, claws with thin pulvilli.

Measurements. Length 6.1mm; width of abdomen across tergite III and IV 3.65mm, V 3.5mm; width of tergite VIII 1.05mm; width /length of tergal plate 2.3/2.1mm; length of antennae 2.35mm.

##### Etymology.

The epithet refers to the piceous colouration of this unusual specimen.

## Supplementary Material

XML Treatment for Osellaptera

XML Treatment for Osellaptera setifera

XML Treatment for Kormilevia

XML Treatment for Kormilevia ecuadoriana

XML Treatment for Cotopaxicoris

XML Treatment for Cotopaxicoris cruciatus

XML Treatment for Onorecoris

XML Treatment for Onorecoris piceus

## References

[B1] Coscaron MC Contreras EF (2012) Catalog of Aradidae (Hemiptera: Heteroptera) for the Neotropical Region. Zootaxa 3466: 1-103

[B2] Douglas JW J Scott (1865) The British Hemiptera. Volume I, Hemiptera-Heteroptera. Ray Society, London, 628 pp.

[B3] Drake CJ (1956) New Neotropical Genera and Species of Apterous Aradids (Hemiptera). Journal of the Washington Academy of Sciences 46: 322-327

[B4] Froeschner RC (1981) Heteroptera or True Bugs of Ecuador: A Partial Catalog. Smithsonian Contributions to Zoology 322: 1-147

[B5] Harris HM Drake CJ (1944) New Apterous Aradidae from the Western Hemisphere Hemiptera). Proceedings of the Entomological Society Washington 46: 128-132

[B6] Kormilev NA (1953a) The First Apterous Aradid from Argentina (Hemiptera). Dusenia 4: 125–126

[B7] Kormilev NA (1953b) Notes on Neotropical Aradidae III (Hemiptera). On some Apterous Mezirinae from Brazil. Dusenia 4: 229-242

[B8] Kormilev NA (1963) Notes on Aradidae in the Naturhistoriska Riksmuseum Stockholm, Hemiptera-Heteroptera. Arkiv för Zoologi 2: 443-455

[B9] Kormilev NA (1964) Neotropical Aradidae XII (Heteroptera: Aradidae). New York Entomological Society 72: 34-39

[B10] Kormilev NA (1983) On the Homonymy of *Hybocoris* Kormilev, 1982 (Hemiptera: Aradidae). Proceedings of the Entomological Society Washington 85: 690

[B11] Kormilev NA Froeschner RC (1987) Flat bugs of the World. A synonymic list. (Heteroptera: Aradidae). Entomography 5: 1-246

[B12] Oshanin BF (1908) Verzeichnis der Palaearktischen Hemipteren mit besonderer Berücksichtigung ihrer Verteilung im Russischen Reiche. 1 (2): 395–586

[B13] Usinger RL (1950) The Origin and Distribution of Apterous Aradidae. Eight International Congress of Entomology, 174–179

[B14] Usinger RL R Matsuda (1959) Classification of the Aradidae. British Museum, London, 410 pp.

[B15] Wygodzinsky P (1948) Studies on some Apterous Aradidae from Brazil (Hemiptera). Boletim do Museo Nacional, Rio de Janeiro, Zoologia 86: 1–23, 24 plates.

